# Decision Fusion with Channel Errors in Distributed Decode-Then-Fuse Sensor Networks

**DOI:** 10.3390/s150819157

**Published:** 2015-08-05

**Authors:** Yongsheng Yan, Haiyan Wang, Xiaohong Shen, Xionghu Zhong

**Affiliations:** 1 School of Marine Science and Technology, Northwestern Polytechnical University, 127 Youyi West Road, Xi’an 710072, China; E-Mails: hywang@nwpu.edu.cn (H.W.); xhshen@nwpu.edu.cn (X.S.); 2 School of Electrical and Electronic Engineering, Nanyang Technological University, 50 Nanyang Avenue, 639798, Singapore; E-Mail: xhzhong@ntu.edu.sg

**Keywords:** decision fusion, channel errors, average bit error rate, likelihood ratio test, decode-then-fuse, sensor networks

## Abstract

Decision fusion for distributed detection in sensor networks under non-ideal channels is investigated in this paper. Usually, the local decisions are transmitted to the fusion center (FC) and decoded, and a fusion rule is then applied to achieve a global decision. We propose an optimal likelihood ratio test (LRT)-based fusion rule to take the uncertainty of the decoded binary data due to modulation, reception mode and communication channel into account. The average bit error rate (BER) is employed to characterize such an uncertainty. Further, the detection performance is analyzed under both non-identical and identical local detection performance indices. In addition, the performance of the proposed method is compared with the existing optimal and suboptimal LRT fusion rules. The results show that the proposed fusion rule is more robust compared to these existing ones.

## 1. Introduction

The advances of wireless communications and distributed signal processes have promoted the deployment of the wireless sensor networks (WSN) in recent years [1,2,3]. Usually, a sensor network employing a number of spatially-distributed sensor nodes is deployed in a surveillance region. These sensor nodes are able to make a decision locally and transmit such local decisions to a fusion center (FC), where a final situation assessment is derived according to the information from local sensors. The capability of detecting a target over a potential region can be significantly enhanced due to the collaborative detection from multiple local sensors. Such a capability, together with the flexibility and scalability of the WSN makes it an attractive solution to many surveillance applications, such as enemy intruder detection [4], health inspection [5], home automation [6] and environment monitoring [7]. Our goal in this paper is to develop a fusion rule at the FC to derive a final decision of the WSN by considering the uncertainty of the received data at the FC due to modulation, reception mode and the communication channel between the FC and sensors.

### 1.1. Related Works and Motivation

**Figure 1:**
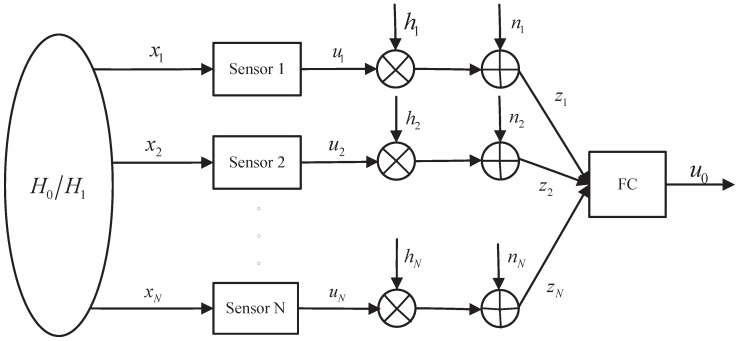
A block diagram of the parallel fusion model in the presence of fading and noisy channels between sensors and the fusion center (FC).

In the past, communication and decision fusion were taken as two separated parts in the application of the typical distributed detection, and the performance analysis for each part was carried out independently. For a distributed detection system with reliable communication, we assume that the local decisions are transmitted to the FC without errors. Based on such an assumption, numerical results on the distributed detection with decision fusion have been widely studied [8,9,10,11,12,13,14]. Under the assumption of conditional independence of the observations, the optimal decision rule at sensors and the optimal fusion rule at the FC are both likelihood-ratio tests (LRTs) [8]. Based on the LRT, several fusion statistics, including Chair–Varshney (CV) [9], generalized LRT [10], the Bayesian fusion statistic [11] and the counting fusion statistic [12,13,14], were proposed. These obtained results rely on the reliable communication between the FC and sensors. However, since the sensors are energy-limited, the transmission power of each sensor is not large enough to make the communication reliable. Usually, the communication channels are subject to channel fading, environmental noise and interferences. For the sake of simplicity, the parallel access channel (PAC) is assumed for information transmission. Each sensor transmits information to the FC across parallel and independent channels. This transmission can be realized through time division multiple access (TDMA), frequency division multiple access (FDMA) or code division multiple access (CDMA). Under the PAC assumption, several channel-aware fusion rules were developed to take the impairment from the channel fading and noise into account [15,16,17,18,19,20]. In [15], an additive white Gaussian noise (AWGN) channel has been considered. A distributed binary detection under an *ad hoc* network was investigated, and a mixed time scale recursive algorithm was proposed. The distributed detection model with fading and noisy channels is illustrated in Figure 1. Each sensor collects raw measurements, makes a local decision and then transmits it to an FC, where the global decision about the presence/absence of a target is given. The fading and noisy channel model considers the physical layer specifications. It can be regarded as a kind of modulation channel, where the waveform carrier modulation and the match-filtering are neglected. Based on this model, an LRT-based channel-aware fusion rule was derived in [17,18]. Starting from the optimal LRT fusion rule, the authors also derived several suboptimal fusion rules: the maximum ratio combiner (MRC), the two-stage CV, and the equal gain combiner (EGC) fusion rules according to the availability of the channel state information (CSI) at the FC. Besides, a unified asymptotic decision rule based on the MRC fusion rule, namely generalized MRC, was proposed to detect weak signals in non-Gaussian noise [19]. These fusion rules significantly rely on the instantaneous CSI. However, acquiring such instantaneous information is cumbersome in resource-constrained applications and is not practical for some time-varying channel. An LRT fusion rule based on channel statistics (LRT-CS) was proposed in [20]. To avoid the requirement of the instantaneous CSI, the authors in [21,22] investigated the impact of imperfect CSI on the fusion rule and detection performance by fixing the total transmission power of training and data symbols. Furthermore, coherent and noncoherent receptions were included in the analysis of the impact of channel uncertainty. Further, decision fusion with multi-hop transmissions [23] was developed to form a channel-aware fusion rule by considering large-scale sensor networks.

These fusion rules based on the physical channel can completely characterize the channel error. However, in a decode-then-fuse system, each sensor observes the noisy measurements and obtains the local decisions according to given decision rules. Then, the decisions are coded, modulated and are transmitted to the FC via the PAC with channel errors. The FC demodulates, decodes the received signal and derives binary results. Then, all of these binary results are combined to give the final detection result about the presence/absence of a target. Based on this model, the received signal is usually decoded first, and a fusion rule is then applied at the FC. Hence, it is the decoded data that determine the final fusion performance. The accuracy of such demodulated data can be characterized by the average bit error rate (BER), which is the final output of the non-ideal PAC. The decode-then-fuse detection system was first given in [24] for MIMO channels. However, it mainly studied the detection performance of the two-stage CV fusion rule, which combines the maximum-likelihood (ML) or minimum mean square error estimate of the transmitted symbols. The optimal fusion rule design in a decode-then-fuse detection system, which combines corrupted binary local decisions that are transmitted from sensors via wireless channels, to the best of our knowledge, is an unaddressed problem. Our goal here is to design the fusion rule to combine corrupted binary local decisions that are transmitted from sensors and to evaluate the detection performance of the decode-then-fuse strategy.

### 1.2. Our Contributions and Paper Organization

Our attempt in this paper is to consider the communication channel errors and the fusion rule as a whole in a decode-then-fuse sensor network. The data uncertainty at the FC due to channel errors, modulation and reception mode are characterized by the BER. The main contributions of this work are summarized as follows.

- An optimal LRT fusion rule with channel errors characterized by BER (LRT-BER) in a decode-then-fuse sensor network is derived. The relation between the decode-then-fuse fusion strategy and the physical layer specifications (modulation, reception mode and channel statistic characteristics of the flat fading communication channels between sensors and the FC) is established.
- The detection performance analysis of the decode-then-fuse sensor network in the presence of channel errors is presented.
- For identical local detection performance indices, the closed-from solution of the system detection performance and threshold choice method are derived by randomized test. For non-identical local detection performance indices, the central limit theorem (CLT) approximation is utilized to perform the performance analysis.

In addition, the detection performance of the proposed fusion rule is compared with the existing fusion rules, e.g., MRC, EGC, two-stage CV and LRT-CS. The simulation results show that better detection performance can be achieved by using the LRT-BER fusion rule.

The rest of this paper is organized as follows. Section 2 gives the detection model and the local decision rule at each sensor. Several previous fusion statistics are reviewed in Section 3. The LRT-BER fusion rule is presented in Section 4. The performance analysis of the proposed fusion rule based on the Neyman–Pearson (N-P) criterion is studied in Section 5. Numerical results are organized in Section 6, and some conclusions are drawn in Section 7.

## 2. Statement of the Problem

**Figure 2:**
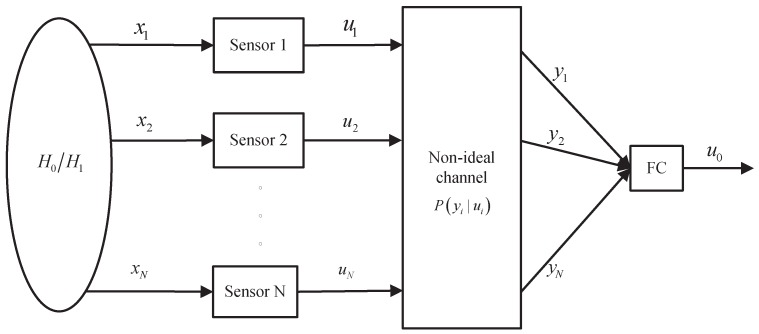
A block diagram of parallel fusion model in the presence of the non-ideal channel characterized by *P*(*y_i_|u_i_*).

Consider an WSN-based target detection system consisting of *N* spatially-distributed sensors and an FC. There are two hypotheses $H_{0}$ and $H_{1}$ under test. $H_{0}$ corresponds to target-absence and $H_{1}$ corresponds to target-presence. Let $X_{i}$ be the observed data at the *i*-th sensor (i=1,⋯,N). Note that $X_{i}$ can be either a random variable or a random vector. The observed data across the whole network are *x* $={\left[X_{1},X_{2},\cdots ,X_{N}\right]}^{T}$, where the superscript *T* denotes the transpose. A local decision rule $\gamma_{i}\left(\cdot \right)$ is applied to the observed data, and a local decision $u_{i}$ is available, *i.e*., $u_{i}=\gamma_{i}\left(X_{i}\right)$, ∀:1≤i≤N. The local decisions can be written in a vector as *u* $={\left[u_{1},u_{2},\cdots ,u_{N}\right]}^{T}$. The local decision $u_{i}$ is coded, modulated and then is transmitted to the FC via a wireless channel with channel errors. The FC decodes, demodulates the received signal and then derives binary corrupted results from sensors. The received data can be distorted by the non-ideal communication channels due to noise and the multipath effect. Furthermore, errors can be brought in by using different modulation/demodulation schemes and reception modes. Assuming that $y_{i}$ is the demodulated data of $u_{i}$ at the FC, the complete impact of the transmission channel, the modulation/demodulation scheme and reception mode can be characterized by $P(y_{i}|u_{i})$. Then, all of these results ***y*** $={\left[y_{1},y_{2},\cdots ,y_{N}\right]}^{T}$ from local sensors are combined according to the proposed fusion rule $\gamma_{0}\left(\cdot \right)$ to give a final detection result $u_{0}$ about the presence/absence of a target, *i.e*., $u_{0}=\gamma_{0}\left(y\right)$. Here, $u_{0}\in \left\{0,1\right\}$ is the final decision of the detection system, where `0’ denotes target absence and `1’ denotes target presence. Figure 2 gives an illustration of such a decision fusion system. It can be shown that the fusion model forms a Markov chain, *i.e*., $H_{0}/H_{1}\to$ ***x*** → ***u*** → ***y*** $\to u_{0}$ is a Markovian process. Since the system detection performance is related to the specifications, including modulation/demodulation schemes, reception modes and the characteristics of the non-ideal communication channel, the more practical BER-based decision fusion strategy for the decode-then-fuse target detection system will be developed and studied in this paper.

To meet the bandwidth limitation of the wireless channel and power-limited budgets of sensors, we consider one-bit quantization according to the local decision rule $u_{i}=\gamma_{i}\left(X_{i}\right)$, *i.e*., $u_{i}\in \left\{0,1\right\}$. We denote $P_{fa_{i}}$ and $P_{d_{i}}$, respectively, as false alarm and detection probabilities corresponding to the local decision $u_{i}$ for the *i*-th sensor. In this paper, we focus on the derivation of the optimal fusion rule at the FC and assume that the local decisions are known, and each sensor detection performance index, the probability of detection and the probability of false alarm, is assumed to be predefined. The local detection performance indices can be determined according to a predefined detection range with a given local decision rule or can be estimated by a recursive algorithm, e.g., joint probability estimation [25].

The proposed fusion strategy is implemented as follows. Each sensor observes noisy measurements and derives a local binary decision. Then, the local decision result is coded and modulated before signal transmission. The fusion strategy in the FC is performed after demodulation and decoding the corrupted decisions from sensors. Thus, the fusion rule can be regarded as a decode-then-fuse one. The main goal of this manuscript is to provide a fusion strategy at the FC based on the demodulated or decoded data. Hence, the intermediate process of the practical modulation/demodulation and coding/decoding is not mentioned. Here, however, we employ “decode” to distinguish the modulation channel model characterized by a flat and slow fading envelope (see Figure 1) that does not consider communication quantization.

## 3. Review of Previous Work

This section gives the modulation channel model characterized by the flat fading envelope $h_{i}$, and the communication quantization is not considered. Several suboptimal fusion statistics based on such a flat fading envelope are reviewed.

Consider a fading and noisy communication channel model (see Figure 1). Each sensor obtains noisy measurements and derives the local decision $u_{i},\;i=1,\cdots ,N$ according to given local decision rules, such as the energy detector and correlation detector. The local decision $u_{i}\in \left\{0,1\right\}$, for i=1,⋯,N is encoded as $b_{i}=2u_{i}-1$ for binary phase-shift keying (BPSK) modulation. Then, each sensor transmits the signal $\rho_{i}\left(t\right)=b_{i}\;\sqrt{E_{i}}\cdot s\left(t\right)$, where $E_{i}$ is the data symbol transmit power and s(t) is a predetermined normalized waveform. At the receiver, *i.e*., the fusion center, the received signal is:

$$\begin{array}{c} z_{i}\left(t\right)=h_{i}\left(t\right)b_{i}\sqrt{E_{i}}\cdot s\left(t\right)+n_{i}\left(t\right) \end{array}$$

where $n_{i}\left(t\right)$ is the channel AWGN with zero mean and variance $\sigma^{2}$ and $h_{i}\left(t\right)$ is a real valued envelope modeled as a Rayleigh distribution with unit second moment gains, *i.e*., $f\left(h_{i}\right)=2h_{i}e^{-h_{i}^{2}}$ for $h_{i}\geq 0$. We assume that the data symbol transmission energy is the unit. After matched filtering by the waveform s(t) at the FC, we have:

(1) $$z_{i}=h_{i}b_{i}+n_{i}$$

The goal is to design the fusion rule according to the data from local sensors. As such, we neglect the waveform carrier modulation and the match-filtering process. The received SNR for the Rayleigh fading envelope with unit second moment gains is defined as SNR $=E\left(h_{i}^{2}\right)/\sigma^{2}=1/\sigma^{2}$, where the E(·) denotes the expectation. For such a distributed detection model, the received data do not consider the communication quantization and take the value between −∞ and +∞. Note that the phase of the channel is assumed to be known, which can be accomplished by shifting the channel estimation from the FC to sensors to pre-compensate for the channel phase rotations [26]. The fusion rule is then applied to obtain a final decision for the target detection system. Here, some widely-used suboptimal fusion statistics based on the flat fading envelope $h_{i}$ in Equation (1) are summarized as follows.

### 3.1. Two-Stage CV Fusion Statistic

Under a high SNR assumption, the received data $z_{i}=h_{i}b_{i}+n_{i}$ at the FC can easily be detected as one or −1. Hence, the optimal LRT rule can be approximated as:

(2) $$\Lambda_{\text{CV}}=\sum_{\text{sign}(z_{i})=1}\log\frac{P_{d_{i}}}{P_{fa_{i}}}+\sum_{\text{sign}(z_{i})=-1}\log\frac{1-P_{d_{i}}}{1-P_{fa_{i}}}$$

The detailed derivation can be found in [20]. It assumes that the channel has high SNRs, and thus, the estimates of the received data are reliable. As the SNR decreases, the approach suffers a significant performance loss.

### 3.2. Maximum Ratio Combiner Fusion Statistic

For the communication channel with low SNRs, it has been shown that the LRT fusion statistic for identical local detection performance indices can be approximated in [18] by:

(3) $$\Lambda_{\text{MRC}}=\frac{1}{N}\sum_{i=1}^{N}h_{i}z_{i}$$

Note that the fusion statistic $\Lambda_{\text{MRC}}$ depends on the channel fading envelope $h_{i}$.

### 3.3. Equal Gain Combiner Fusion Statistic

Motivated by the MRC statistic, the EGC fusion statistic, which requires the minimum amount of prior information about the communication channel, is also proposed in [18], expressed as:

(4) $$\Lambda_{\mathrm{EGC}}=\frac{1}{N}\sum_{i=1}^{N}z_{i}$$

Note that neither the knowledge of the communication channel nor the local detection performance indices $P_{d_{i}}$ and $P_{fa_{i}}$ are required for the statistic $\Lambda_{\text{EGC}}$.

### 3.4. LRT Based on Channel Statistics

In [20], a new LRT fusion rule based on the prior information regarding the channel statistic characteristics instead of the instantaneous CSI $h_{i}$ is proposed. Assuming a Rayleigh fading channel with unit power, *i.e*., $f\left(h_{i}\right)=2h_{i}e^{-h_{i}^{2}},h_{i}\geq 0$, the LRT-CS can be written as:

(5) $$\begin{array}{cc} & \Lambda_{\mathrm{CS}}=\sum_{i=1}^{N}\log\frac{1+\left(P_{d_{i}}-Q\left(az_{i}\right)\right)\sqrt{2\pi }az_{i}e^{\frac{{\left(az_{i}\right)}^{2}}{2}}}{1+\left(P_{fa_{i}}-Q\left(az_{i}\right)\right)\sqrt{2\pi }az_{i}e^{\frac{{\left(az_{i}\right)}^{2}}{2}}} \end{array}$$

where Q(·) is the complementary distribution function of the standard Gaussian expressed as $Q\left(x\right)=\frac{1}{2\pi }\int_{x}^{\infty }e^{-\frac{y^{2}}{2}}dy$ and $a=1/(\sigma \sqrt{1+2\sigma^{2}})$. The LRT-CS incorporates the Rayleigh fading characteristics into the fusion statistic to avoid the requirement of the instantaneous CSI.

## 4. Decision Fusion Rule with Channel Errors Characterized by BER

This section gives the optimal fusion rule based on the non-ideal channel characterized by the probability $P(y_{i}|u_{i})$. It can be written as a function of BER. The BER is able to describe the data uncertainty at the FC due to channel errors, as well as modulations/demodulations. By taking different physical layer specifications, including modulation, reception mode and channel characteristics between sensors and the FC, the BER is derived. In this paper, we consider the flat and slow fading channel. This means that the channel fading is roughly equal across the entire signal bandwidth for a narrowband signal transmission [27].

For a decode-then-fuse distributed detection system, the FC decodes the received data and obtains the binary quantization results. Thus, the wireless and non-ideal channel can be modeled as the probability $P(y_{i}|u_{i})$. The probability of a *k* bit error, when *n* bit data are transmitted, follows a binomial distribution, given as [28]:

(6) P(n,k)=nk(Pe)k(1−Pe)(n−k)

where $P_{e}$ is the channel BER. We have $P\left(y_{i}=0|u_{i}=1\right)=P\left(y_{i}=1|u_{i}=0\right)=P\left(1,1\right)=P_{e}$. P(1,1) denotes the probability of one-bit error for one-bit transmission. As we know, the BER depends on several factors of the communication channel, including the reception mode at the FC, the signal modulation/demodulation method at sensors and the FC and the statistic characteristics of the non-ideal channel. Thus, the overall impact from the non-ideal channel and the different modulation/demodulation schemes on the received data can be characterized by $P_{e}$. As such, the fusion rule of the decode-then-fuse collaborative detection system and the physical layer specifications are related to each other by $P_{e}$.

For a flat and slow Rayleigh fading channel, the BER with coherent binary frequency-shift keying (BFSK or 2FSK) modulation, coherent BPSK modulation and noncoherent differential phase-shift keying (DPSK) modulation can be addressed as the following relations (see [27] pp: 170∼176):

(7) $$\begin{array}{c} P_{e}=\left\{\begin{array}{c} \frac{1}{2}\left(1-\sqrt{\frac{{\bar{\gamma }}_{b}}{2+{\bar{\gamma }}_{b}}}\right)\;\text{coherent}\;\text{2FSK}\;\text{modulation}\\ \frac{1}{2}\left(1-\sqrt{\frac{{\bar{\gamma }}_{b}}{1+{\bar{\gamma }}_{b}}}\right)\;\text{coherent}\;\text{BPSK}\;\text{modulation}\\ \frac{1}{2\left(1+{\bar{\gamma }}_{b}\right)}\;\text{noncoherent}\;\text{DPSK}\;\text{modulation} \end{array}\right. \end{array}$$

where ${\bar{\gamma }}_{b}$ is the average received SNR of the communication channel, defined as:

(8) $$\begin{array}{c} {\bar{\gamma }}_{b}=\frac{P_{r}}{N_{0}B}=\frac{P_{r}}{\sigma^{2}} \end{array}$$

where $N_{0}B=N_{0}/2\times 2B$ is the total noise power within the bandwidth 2B and $\sigma^{2}=N_{0}/2\times 2B$. $P_{r}$ is the received signal power. Furthermore, the received SNR can be regarded as ${\bar{\gamma }}_{b}=E_{b}/\left(N_{0}BT_{b}\right)=E_{b}/N_{0}$ for data pulses with $T_{b}=1/B$ [27]. For a flat and slow Ricean fading channel with different *K* factors, the BER with coherent 2FSK modulation, coherent BPSK modulation and noncoherent DPSK modulation can be written as:

(9) $$\begin{array}{c} P_{e}=\left\{\begin{array}{c} \frac{1}{\pi }\int_{0}^{\frac{\pi }{2}}\frac{\left(1+K\right)\sin^{2}\phi }{\left(1+K\right)\sin^{2}\phi +0.5{\bar{\gamma }}_{b}}\exp\left(-\frac{0.5K{\bar{\gamma }}_{b}}{\left(1+K\right)\sin^{2}\phi +0.5{\bar{\gamma }}_{b}}\right)d\phi \\ \text{coherent}\;\text{2FSK}\;\text{modulation}\\ \frac{1}{\pi }\int_{0}^{\frac{\pi }{2}}\frac{\left(1+K\right)\sin^{2}\phi }{\left(1+K\right)\sin^{2}\phi +{\bar{\gamma }}_{b}}\exp\left(-\frac{K{\bar{\gamma }}_{b}}{\left(1+K\right)\sin^{2}\phi +{\bar{\gamma }}_{b}}\right)d\phi \\ \text{coherent}\;\text{BPSK}\;\text{modulation}\\ \frac{1+K}{2(1+K+{\bar{\gamma }}_{b})}\exp\left(-\frac{K{\bar{\gamma }}_{b}}{1+K+{\bar{\gamma }}_{b}}\right)\\ \text{noncoherent}\;\text{DPSK}\;\text{modulation} \end{array}\right. \end{array}$$

where *K* is the ratio of the powers of the line of sight component to the scattered component of the received signal. For a flat and slow Nakagami-*m* fading channel, the BER with coherent 2FSK modulation, coherent BPSK modulation and noncoherent DPSK modulation is given as:

(10) $$\begin{array}{c} P_{e}=\left\{\begin{array}{c} \frac{1}{\pi }\int_{0}^{\frac{\pi }{2}}{\left(1+\frac{0.5{\bar{\gamma }}_{b}}{m\sin^{2}\phi }\right)}^{-m}d\phi \;\text{coherent}\;\text{2FSK}\;\text{modulation}\\ \frac{1}{\pi }\int_{0}^{\frac{\pi }{2}}{\left(1+\frac{{\bar{\gamma }}_{b}}{m\sin^{2}\phi }\right)}^{-m}d\phi \;\text{coherent}\;\text{BPSK}\;\text{modulation}\\ \frac{1}{2}\int_{0}^{\frac{\pi }{2}}{\left(1+\frac{{\bar{\gamma }}_{b}}{m}\right)}^{-m}d\phi \;\text{noncoherent}\;\text{DPSK}\;\text{modulation} \end{array}\right. \end{array}$$

where *m* is the Nakagami parameter. The detailed derivation of these BERs can be found in [27].

The main task of this paper is to construct a fusion rule according to the decoded results $y_{i}$, for 1≤i≤N. Here, we only consider the N-P criterion, since the prior probability of the hypotheses is usually unknown *a priori*. The optimal LRT-based fusion rule for N-P criterion can be written as:

(11) $$\frac{P(y|H_{1})}{P(y|H_{0})}\left.\begin{array}{c} \overset{H_{1}}{\geq }\\ \underset{H_{0}}{<} \end{array}\right.T_{N}$$

where $T_{N}$ is the fusion threshold of the LRT fusion statistic. The LRT statistic (11) based on the knowledge of channel BER can be further written as:

(12) $$\begin{array}{cc} \Lambda = & \frac{P\left(y|H_{1}\right)}{P\left(y|H_{0}\right)}=\prod_{i=1}^{N}\frac{P(y_{i}|H_{1})}{P(y_{i}|H_{0})}\\ = & \prod_{i=1}^{N}\frac{\sum_{u_{i}}P\left(y_{i}|u_{i}\right)P\left(u_{i}|H_{1}\right)}{\sum_{u_{i}}P\left(y_{i}|u_{i}\right)P\left(u_{i}|H_{0}\right)}\\ = & \prod_{i=1}^{N}\frac{P\left(y_{i}|u_{i}=1\right)P_{d_{i}}+P\left(y_{i}|u_{i}=0\right)\left(1-P_{d_{i}}\right)}{P\left(y_{i}|u_{i}=1\right)P_{fa_{i}}+P\left(y_{i}|u_{i}=0\right)\left(1-P_{fa_{i}}\right)} \end{array}$$

where $P_{d_{i}}$ is the detection probability and $P_{fa_{i}}$ is the false alarm probability of the *i*-th sensor. The above equation is obtained by following the fact that $H_{0}/H_{1}\to$ ***x*** → ***u*** → ***y*** $\to u_{0}$ forms a Markov chain in Figure 2. The local binary decision results are transmitted by using the BPSK, 2FSK or DPSK modulation scheme. The demodulated data at the FC are also binary, *i.e*., $y_{i}\in \left\{0,1\right\}$. Expanding the fusion statistic Λ in Equation (12) with respect to the one-bit error probability, we have:

(13) $$\begin{array}{c} \Lambda =\prod_{i=1}^{N}\frac{{\left(1-P_{e}\right)}^{y_{i}}P_{e}^{\left(1-y_{i}\right)}P_{d_{i}}+P_{e}^{y_{i}}{\left(1-P_{e}\right)}^{\left(1-y_{i}\right)}\left(1-P_{d_{i}}\right)}{{\left(1-P_{e}\right)}^{y_{i}}P_{e}^{\left(1-y_{i}\right)}P_{fa_{i}}+P_{e}^{y_{i}}{\left(1-P_{e}\right)}^{\left(1-y_{i}\right)}\left(1-P_{fa_{i}}\right)} \end{array}$$

Note that in Equation (13), the equation holds $y_{i}$ is either zero or one, and the facts $P\left(y_{i}=0|u_{i}=1\right)=P\left(y_{i}=1|u_{i}=0\right)=P_{e}$ and $P\left(y_{i}=1|u_{i}=1\right)=P\left(y_{i}=0|u_{i}=0\right)=1-P_{e}$ according to Equation (6) are employed. For Equation (13), two cases ($P_{e}=0$ and $0\leq P_{e}\leq 1/2$) are considered as follows.

(1) If $P_{e}=0$, *i.e*., the communication channel is error-free, $\Lambda_{i}$ in the BER-based fusion statistic $\Lambda =\prod_{i=1}^{N}\Lambda_{i}$ can be reduced to:
  (14) $$\Lambda_{i}=\left\{\begin{array}{cc} \frac{P_{d_{i}}}{P_{fa_{i}}}, & y_{i}=1\left(\text{or}\;u_{i}=1\right),P_{e}=0\\ \frac{1-P_{d_{i}}}{1-P_{fa_{i}}}, & y_{i}=0\left(\text{or}\;u_{i}=0\right),P_{e}=0 \end{array}\right.$$
  This fusion statistic is the CV statistic in [9]. Note that the CV statistic instead of the two-stage CV statistic is the optimal fusion statistic under error-free communication channels.
(2) If $0<P_{e}\leq 1/2$, *i.e*., the local decisions are subject to transmission errors, the fusion statistic (13) can be written as:
  (15) $$\Lambda =\prod_{i=1}^{N}\frac{P_{d_{i}}+{P_{e}}^{2y_{i}-1}{\left(1-P_{e}\right)}^{1-2y_{i}}\left(1-P_{d_{i}}\right)}{P_{fa_{i}}+{P_{e}}^{2y_{i}-1}{\left(1-P_{e}\right)}^{1-2y_{i}}\left(1-P_{fa_{i}}\right)}$$

It can be observed that the fusion statistic for the setup $P_{e}=0$ can be regarded as the CV statistic under the ideal transmission channels. Here, we focus on the channel-aware fusion rule, and the fusion statistic with the $0<P_{e}\leq 1/2$ setup is considered. Taking the logarithm of Equation (15), we obtain the log-likelihood ratio based on BER (LRT-BER), shown as:

(16) $$\Lambda_{\text{BER}}=\sum_{i=1}^{N}\log\left[\frac{P_{d_{i}}+{\left(P_{e}/\left(1-P_{e}\right)\right)}^{2y_{i}-1}\left(1-P_{d_{i}}\right)}{P_{fa_{i}}+{\left(P_{e}/\left(1-P_{e}\right)\right)}^{2y_{i}-1}\left(1-P_{fa_{i}}\right)}\right]$$

Let $S_{0}=\left\{i:y_{i}=0\right\}$ and $S_{1}=\left\{i:y_{i}=1\right\}$. Alternatively, Equation (16) can be further expressed as:

(17) $$\begin{array}{cc} \Lambda_{\text{BER}}= & \sum_{i\in S_{1}}\log\frac{P_{d_{i}}+\left(P_{e}/\left(1-P_{e}\right)\right)\left(1-P_{d_{i}}\right)}{P_{fa_{i}}+\left(P_{e}/\left(1-P_{e}\right)\right)\left(1-P_{fa_{i}}\right)}\\ & +\sum_{i\in S_{0}}\log\frac{P_{d_{i}}+\left(\left(1-P_{e}\right)/P_{e}\right)\left(1-P_{d_{i}}\right)}{P_{fa_{i}}+\left(\left(1-P_{e}\right)/P_{e}\right)\left(1-P_{fa_{i}}\right)} \end{array}$$

The BER-based fusion statistic $\Lambda_{\text{BER}}$ requires the knowledge of the sensor detection probability $P_{d_{i}}$, the false alarm probability $P_{fa_{i}}$ and the BER of the communication channel between each sensor and the FC. The fusion statistic is based on the assumption that the transmitted symbol is {0,1}. It is straightforward to show that the symbol {+1,−1} leads to an identical fusion statistic with the symbol {0,1}. For the proposed BER-based fusion rule, the modulation mode is reflected in the BER rather than the transmission symbol. By contrast, for the flat and slow fading channel characterized by the channel envelope $h_{i}$, the modulation is reflected in the transmission symbols ({+1,−1} and {0,1}) [21,22].

Notice that, for the decode-then-fuse strategy Equation (17), the communication channel between sensor *i* and the FC is modeled as the probability $P(y_{i}|u_{i})$ for binary $u_{i}$ and $y_{i}$. Such a probability can completely characterize the uncertainties due to modulation/demodulation, reception mode, as well as the channel flat fading statistics’ characteristics. As such, we can focus on the fusion process, and the communication errors between sensors and the fusion center are taken into account by the probability $P(y_{i}|u_{i})$, which can be expressed as the function of $P_{e}$. $P_{e}$ in Equations (7), (9) and (10) is a function of the received SNR ${\bar{\gamma }}_{b}$, which can be estimated by transmitting training symbols before fusing data transmission [29,30,31].

## 5. Performance Analysis and Discussion

The system detection performance of the proposed LRT-BER fusion rule is evaluated in this section. Here, we consider two cases: (1) sensors with identical local detection performance, *i.e*., the detection and false alarm probabilities are equal; and (2) sensors with different detection performance. For the former case, the closed-form performance analysis is carried out by deriving the distribution of the fusion statistic LRT-BER. For the latter case, the closed-form solutions are difficult to derive, since the distribution of the $\Lambda_{\text{BER}}$ fusion statistic is a nonlinear function of $y_{i}$. The central limit theorem (CLT) and the deflection coefficient are employed to carry out the performance evaluation.

### 5.1. Closed-Form Performance Analysis for Identical Local Detection Performance Indices

If the local detection performance indices are equal to each other, *i.e*., $P_{d_{i}}=P_{d}$ and $P_{fa_{i}}=P_{fa}$, for i=1,⋯,N, the fusion statistic Equation (17) can be written as:

(18) $$\begin{array}{cc} \Lambda_{\text{BER}}^{\prime } & =N_{1}^{\prime }\log\frac{P_{d}+P_{e}/\left(1-P_{e}\right)\left(1-P_{d}\right)}{P_{fa}+P_{e}/\left(1-P_{e}\right)\left(1-P_{fa}\right)}\\ + & \underset{N_{0}^{\prime }}{\underset{︸}{\left(N-N_{1}^{\prime }\right)}}\log\frac{P_{d}+\left(1-P_{e}\right)/P_{e}\left(1-P_{d}\right)}{P_{fa}+\left(1-P_{e}\right)/P_{e}\left(1-P_{fa}\right)}\\ & =N_{1}^{\prime }C_{1}+C_{2} \end{array}$$

where $N_{0}^{\prime }=\left|S_{0}\right|$ and $N_{1}^{\prime }=\left|S_{1}\right|$ with |·| denoting the cardinality, that is $N_{0}^{\prime }$ is the number of $y_{i}=0$ in all *N* demodulated data, whereas $N_{1}^{\prime }$ is the number of $y_{i}=1$ in all *N* demodulated data. $C_{1}$ and $C_{2}$ are constants given as:

(19) $$\begin{array}{cc} C_{1}=\log & \left[\frac{P_{d}+P_{e}/\left(1-P_{e}\right)\left(1-P_{d}\right)}{P_{fa}+P_{e}/\left(1-P_{e}\right)\left(1-P_{fa}\right)}\right.\\ & \cdot \left.\frac{P_{fa}+\left(1-P_{e}\right)/P_{e}\left(1-P_{fa}\right)}{P_{d}+\left(1-P_{e}\right)/P_{e}\left(1-P_{d}\right)}\right] \end{array}$$

and:

(20) $$C_{2}=N\log\left[\frac{P_{d}+\left(1-P_{e}\right)/P_{e}\left(1-P_{d}\right)}{P_{fa}+\left(1-P_{e}\right)/P_{e}\left(1-P_{fa}\right)}\right]$$

The fusion statistic $\Lambda_{\text{BER}}^{\prime }$ is an affine function of $N_{1}^{\prime }$. Under both hypotheses $H_{1}/H_{0}$, $N_{1}^{\prime }=\sum_{i=1}^{N}y_{i}$ has a binomial distribution, *i.e*., $N_{1}^{\prime }∼B\left(N,P_{j}\right)$, for j∈{0,1}. Here, the success probability under $H_{1}$ hypothesis is:

(21) $$P_{1}=P\left(y_{i}=1|H_{1}\right)=\left(1-P_{e}\right)P_{d}+P_{e}\left(1-P_{d}\right)$$

Similarly, the probability of $y_{i}=1$ under $H_{0}$ hypothesis is:

(22) $$P_{0}=P\left(y_{i}=1|H_{0}\right)=\left(1-P_{e}\right)P_{fa}+P_{e}\left(1-P_{fa}\right)$$

As such, under the N-P criterion, given a predefined system false alarm probability *α*, we have:

(23) PFA=∑k∈κNkP0k(1−P0)N−k≤α

where $\kappa =\{k:\;k=\sum_{i=1}^{N}y_{i}\geq T\}$ and *T* represents the fusion threshold of the statistic $N_{1}^{\prime }$. The threshold *T* can be obtained by maximizing all of the solutions that satisfy Inequality in Equation (23). Correspondingly, the detection probability of the fusion statistic $N_{1}^{\prime }$ can be written as:

(24) PD=Pr(N1′=k≥T|H1)=∑k∈κNkP1k(1−P1)N−k

For the fusion statistic $\Lambda_{\text{BER}}^{\prime }=N_{1}^{\prime }C_{1}+C_{2}$, when $C_{1}>0$, *i.e*., $\left(P_{d}-P_{fa}\right)\left(1-2P_{e}\right)>0$, the detection probability of $\Lambda_{\mathrm{BER}}^{\prime }$ is:

(25) $$\begin{array}{cc} P_{\text{D}}^{\prime } & =\mathrm{Pr}(N_{1}^{\prime }C_{1}+C_{2}\geq T^{\prime }|H_{1})\\ & =\mathrm{Pr}(N_{1}^{\prime }\geq \frac{T^{\prime }-C_{2}}{C_{1}}|H_{1})\\ & \;≜\mathrm{Pr}(N_{1}^{\prime }\geq T|H_{1})\\ & =P_{\text{D}} \end{array}$$

where $T^{\prime }$ is the threshold of $\Lambda_{\text{BER}}^{\prime }$. The condition $\left(P_{d}-P_{fa}\right)\left(1-2P_{e}\right)>0$ is satisfied when the detection probability $P_{d}$ is larger than the false alarm probability $P_{fa}$ and the BER $P_{e}$ is less than 1/2. This condition is easily satisfied for a practical decode-then-fuse target detection system. It can be observed from Equations (24) and (25) that the detection probability is equal for the fusion statistic $N_{1}^{\prime }$ and $\Lambda_{\text{BER}}^{\prime }$. The constants $C_{1}$ and $C_{2}$ will lead to different thresholds for $N_{1}^{\prime }$ and $\Lambda_{\text{BER}}^{\prime }$. For the statistic $N_{1}^{\prime }$, the threshold is *T*, while for the statistic $\Lambda_{\text{BER}}^{\prime }$, the threshold is $T^{\prime }=C_{1}T+C_{2}$.

According to the N-P criterion, it is required to maximize the system detection probability of the FC while the system false alarm probability is below a given value *α*. Note that the threshold *T* only takes values from zero to *N*. For such a case with finite integer numbers of the fusion threshold, the desired system false alarm probability *α* cannot be achieved. Hence, one can use the randomized fusion strategy under the N-P criterion. Under such a strategy, the FC declares the “detect” randomly with a probability [32,33]. The fusion space can be partitioned into three parts, given as:

(26) $$\begin{array}{c} \delta \left(y\right)=\left\{\begin{array}{cc} 1, & \Lambda_{\text{BER}}^{\prime }\left(y\right)>T_{\text{R}}\\ \omega , & \Lambda_{\text{BER}}^{\prime }\left(y\right)=T_{\text{R}}\\ 0, & \Lambda_{\text{BER}}^{\prime }\left(y\right)<T_{\text{R}} \end{array}\right. \end{array}$$

where *ω*, 0≤ω≤1 is a randomized factor and $T_{\text{R}}$ is the randomized fusion threshold. The procedure to obtain the fusion threshold $T_{\text{R}}$ and the randomized factor *ω* is summarized as follows.

(1) Search and obtain the randomized fusion thresholds ***T*_R_** $={\left[T_{\text{R}}^{1},\cdots ,T_{\text{R}}^{L}\right]}^{T}$ to satisfy the inequality
  (27) ∑k∈κRNkP0k(1−P0)N−k≤α
  where $\kappa_{\text{R}}$ is defined as $\kappa_{\text{R}}=\left\{k:\;k=\sum_{i=1}^{N}y_{i}\geq T_{\text{R}}^{l}\right\}$, 1≤l≤L. *L* is the number of thresholds that satisfy the inequality.
(2) Maximize the searching thresholds ***T***_R_ in step 1 to obtain *T*_R_, *i.e.*, *T*_R_ = max($T_{\text{R}}$).
(3) Obtain the randomized factor ω by:
  (28) $$\omega =\frac{\alpha -\sum {}_{k\in \kappa_{\text{R}}}\left(\begin{array}{c} N\\ k \end{array}\right)P_{0}^{k}{\left(1-P_{0}\right)}^{N-k}}{\left(\begin{array}{c} N\\ T_{\text{R}} \end{array}\right)P_{0}^{T_{\text{R}}}{\left(1-P_{0}\right)}^{N-T_{\text{R}}}}$$
(4) The system false alarm probability $P_{\mathrm{FA}}^{\text{R}}$ for randomized detection can be written as:
  (29) PFAR=α=∑k∈κRNkP0k(1−P0)N−k+ωNTRP0TR(1−P0)N−TR

Similarly, the system detection probability $P_{\text{D}}^{\text{R}}$ is:

(30) PDR=∑k∈κRNkP1k(1−P1)N−k+ωNTRP1TR(1−P1)N−TR

For randomized fusion strategy, the given false alarm probability α can be achieved by adjusting the term ωNTRP0TR(1−P0)N−TR under the N-P criterion. However, for the non-randomized fusion rule in [18], there always exists a gap between the required system false alarm probability α and $P_{\mathrm{FA}}$.

### 5.2. Asymptotic Performance Analysis for Identical Local Detection Performance Indices

In this subsection, the asymptotic performance analysis with respect to a large number of sensors is carried out by using the CLT approximation when the local detection performance indices are equal to each other. The fusion statistic $\Lambda_{\text{BER}}^{\prime }$ is equivalent to the statistic $N_{1}^{\prime }$ in terms of the system detection performance. $N_{1}^{\prime }$ follows the binomial distribution. For the CLT approximation performance evaluation, the system false alarm probability and detection probability can be written as:

(31) $$P_{\mathrm{FA}}=P\left(\Lambda_{\text{BER}}^{\prime }\geq T_{\text{C}}|H_{0}\right)=Q\left(\frac{T_{\text{C}}-NP_{0}}{\sqrt{NP_{0}\left(1-P_{0}\right)}}\right)$$

and:

(32) $$P_{D}=P\left(\Lambda_{\text{BER}}^{\prime }\geq T_{\text{C}}|H_{1}\right)=Q\left(\frac{T_{\text{C}}-NP_{1}}{\sqrt{NP_{1}\left(1-P_{1}\right)}}\right)$$

where $P_{1}$ and $P_{0}$ are given in Equations (21) and (22). $T_{\text{C}}$ is the fusion threshold under this case. Q(·) is the complementary distribution function of the standard Gaussian. Clearly, $NP_{0}$ and $NP_{0}\left(1-P_{0}\right)$ are the mean and variance under $H_{0}$ hypothesis. Similarly, $NP_{1}$ and $NP_{1}\left(1-P_{1}\right)$ are the mean and variance under $H_{1}$ hypothesis. The CLT approximation does have a tractable performance evaluation. From Equations (31) and (32), we have:

$$\begin{array}{c} P_{D}=Q\left(\frac{Q^{-1}\left(P_{\mathrm{FA}}\right)\sqrt{P_{0}\left(1-P_{0}\right)}-\sqrt{N}\left(P_{1}-P_{0}\right)}{\sqrt{P_{1}\left(1-P_{1}\right)}}\right) \end{array}$$

According to the N-P criterion, it is required to maximize the system detection probability at the FC while the system false alarm probability is below a given value α. When:

$$\begin{array}{c} P_{1}-P_{0}=\left(1-2P_{e}\right)\left(P_{d}-P_{fa}\right)>0 \end{array}$$

*i.e*., $P_{1}>P_{0}$, under identical system alarm probability $\alpha =P_{\mathrm{FA}}$, the system detection probability $P_{D}$ can be enhanced when the number of sensor N increases due to the monotonic decreasing characteristic of function Q(·).

### 5.3. Asymptotic Performance Analysis for Non-Identical Local Detection Performance Indices

For non-identical local detection performance indices, *i.e*., $P_{d_{i}}\neq P_{d_{j}}$ for i≠j, the closed-form expression for the system detection performance is difficult to derive. Here, we resort to the asymptotic performance analysis. The statistic $\Lambda_{\text{BER}}$ is the sum of independent random variables. This allows direct application of the CLT approximation, and the limiting distribution under the $H_{0}$ and $H_{1}$ hypotheses is Gaussian with respective mean and variance. The mean and variance under the $H_{1}$ hypothesis are:

(33) $$\begin{array}{cc} E(\Lambda_{\text{BER}}|H_{1})= & \sum_{i=1}^{N}[g\left(y_{i}=1\right)\left(\left(1-P_{e}\right)P_{d_{i}}+P_{e}\left(1-P_{d_{i}}\right)\right)\\ & \;\;\;\;+g\left(y_{i}=0\right)\left(P_{e}P_{d_{i}}+\left(1-P_{e}\right)\left(1-P_{d_{i}}\right)\right)]\\ \overset{\Delta }{=} & \sum_{i=1}^{N}f\left(y_{i},P_{d_{i}}\right)\overset{\Delta }{=}\mu_{\text{BER1}} \end{array}$$

and:

(34) $$\begin{array}{cc} V_{\text{ar}}\left(\Lambda_{\text{BER}}|H_{1}\right) & =\sum_{i=1}^{N}\left[E\left(g^{2}\left(y_{i}\right)|H_{1}\right)-f^{2}\left(y_{i},P_{d_{i}}\right)\right]\\ & \overset{\Delta }{=}\sigma_{\text{BER1}}^{2} \end{array}$$

where $g(y_{i})$ is defined as:

(35) $$g\left(y_{i}\right)=\log\left[\frac{P_{d_{i}}+{\left(P_{e}/\left(1-P_{e}\right)\right)}^{2y_{i}-1}\left(1-P_{d_{i}}\right)}{P_{{\mathrm{fa}}_{i}}+{\left(P_{e}/\left(1-P_{e}\right)\right)}^{2y_{i}-1}\left(1-P_{{\mathrm{fa}}_{i}}\right)}\right]$$

and $f(y_{i},P_{d_{i}})$ is defined as:

(36) $$\begin{array}{cc} f(y_{i},P_{d_{i}}) & =g\left(y_{i}=1\right)\left(\left(1-P_{e}\right)P_{d_{i}}+P_{e}\left(1-P_{d_{i}}\right)\right)\\ + & g\left(y_{i}=0\right)\left(P_{e}P_{d_{i}}+\left(1-P_{e}\right)\left(1-P_{d_{i}}\right)\right) \end{array}$$

$E(g^{2}\left(y_{i}\right)|H_{1})$ denotes:

(37) $$\begin{array}{cc} E(g^{2}\left(y_{i}\right) & |H_{1})=g^{2}\left(y_{i}=1\right)\left(\left(1-P_{e}\right)P_{d_{i}}+P_{e}\left(1-P_{d_{i}}\right)\right)\\ & +g^{2}\left(y_{i}=0\right)\left(P_{e}P_{d_{i}}+\left(1-P_{e}\right)\left(1-P_{d_{i}}\right)\right) \end{array}$$

Similarly, the mean and variance under the $H_{0}$ hypothesis are:

(38) $$E(\Lambda_{\text{BER}}|H_{0})\overset{\Delta }{=}\sum_{i=1}^{N}f\left(y_{i},P_{{\mathrm{fa}}_{i}}\right)\overset{\Delta }{=}\mu_{\text{BER0}}$$

and:

(39) $$\begin{array}{cc} V_{\text{ar}}\left(\Lambda_{\text{BER}}|H_{0}\right) & =\sum_{i=1}^{N}\left[E\left(g^{2}\left(y_{i}\right)|H_{0}\right)-f^{2}\left(y_{i},P_{{\mathrm{fa}}_{i}}\right)\right]\\ & \overset{\Delta }{=}\sigma_{\text{BER0}}^{2} \end{array}$$

where $E(g^{2}\left(y_{i}\right)|H_{0})$ is defined as:

(40) $$\begin{array}{cc} E(g^{2}\left(y_{i}\right)|H_{0})= & g^{2}\left(y_{i}=1\right)(\left(1-P_{e}\right)P_{{\mathrm{fa}}_{i}}+P_{e}\left(1-P_{{\mathrm{fa}}_{i}}\right))\\ & +g^{2}\left(y_{i}=0\right)\left(P_{e}P_{{\mathrm{fa}}_{i}}+\left(1-P_{e}\right)\left(1-P_{{\mathrm{fa}}_{i}}\right)\right) \end{array}$$

By using the CLT approximation, the system false alarm probability can be written as:

(41) $$\begin{array}{c} P_{\mathrm{FA}}=P\left\{\Lambda_{\text{BER}}\geq \Gamma |H_{0}\right\}=Q\left(\frac{\Gamma -\mu_{\text{BER0}}}{\sigma_{\text{BER0}}}\right) \end{array}$$

where Γ is the threshold of the fusion statistic. The corresponding detection probability is:

(42) $$\begin{array}{cc} P_{\text{D}} & =P\left\{\Lambda_{\text{BER}}\geq \Gamma |H_{1}\right\}=Q\left(\frac{\Gamma -\mu_{\text{BER1}}}{\sigma_{\text{BER1}}}\right) \end{array}$$

It is interesting to find that the accuracy of CLT approximation depends not only on the number of sensors, but also on the local detection performance indices and received SNRs [18].

### 5.4. Deflection Coefficient Performance Analysis

In addition to the CLT approximation, an alternative performance analysis can be made through the deflection coefficient [34,35]. The optimal detection performance of the LRT-based fusion rule can be obtained by maximizing the deflection coefficient when a Gaussian signal in Gaussian noise is detected [20]. The deflection coefficient is defined as:

(43) $$D\left(\Lambda \right)=\frac{{\left[E\left(\Lambda |H_{1}\right)-E\left(\Lambda |H_{0}\right)\right]}^{2}}{V_{\mathrm{ar}}\left(\Lambda |H_{0}\right)}$$

where $E\left(\cdot |H_{j}\right),j\in \left\{0,1\right\}$ is the mean under $H_{j}$ hypothesis and $V_{\mathrm{ar}}\left(\cdot |H_{j}\right)$ is the variance under $H_{j}$ hypothesis.

From Equation (43), when the local detection performance indices are equal to each other, the deflection coefficient is given as:

(44) $$\begin{array}{cc} d_{1}^{2}= & \frac{{\left[E\left(\Lambda_{\text{BER}}^{\prime }|H_{1}\right)-E\left(\Lambda_{\text{BER}}^{\prime }|H_{0}\right)\right]}^{2}}{V_{\text{ar}}\left(\Lambda_{\text{BER}}^{\prime }|H_{0}\right)}\\ = & \frac{N{\left(P_{1}-P_{0}\right)}^{2}}{P_{0}\left(1-P_{0}\right)} \end{array}$$

Similarly, when the local detections of the deflection performance indices are not equal, the deflection coefficient is given as:

(45) $$d_{2}^{2}=\frac{{\left[E\left(\Lambda_{\text{BER}}|H_{1}\right)-E\left(\Lambda_{\text{BER}}|H_{0}\right)\right]}^{2}}{V_{\text{ar}}\left(\Lambda_{\text{BER}}|H_{0}\right)}$$

where $E(\Lambda_{\text{BER}}|H_{1})$ and $E(\Lambda_{\text{BER}}|H_{0})$ are the means given in Equations (33) and (38), respectively. $V_{\text{ar}}\left(\Lambda_{\text{BER}}|H_{0}\right)$ is the variance given in Equation (39). Unlike the closed-form performance analysis that needs the distribution of the fusion statistic, the deflection coefficient only requires the first and the second order moments under the $H_{1}/H_{0}$ hypothesis.

### 5.5. Practical Issues

The PAC is assumed for MRC, EGC, two-stage CV and the proposed LRT-BER fusion rule. When each sensor simultaneously transmits a signal to the FC via such a PAC, which can be realized through frequency division multiple access, the bandwidth requirement for the sensor network is large, especially for the large-scale sensor network. When each sensor transmits a signal by TDMA, a large detection delay is required. The bandwidth requirement or detection delay is equal for identical scale networks.

Under the PAC assumption, the type-based distributed detection strategies are proposed in [36,37], where the power consumption for signal transmission is established. The same power consumption model can be employed in the LRT-BER fusion rule, which is given as:

(46) $$P_{\text{E}}=\sum_{i=1}^{N}E\left[E_{i}\cdot {\left(u_{i}\right)}^{2}\right]$$

where E[·] denotes the expectation and $E_{i}$ denotes data symbol transmit power. Note that all of these fusion rules are based on the PAC assumption. Thus, the power consumption for signal transmission are identical for all of these fusion rules. Further, the proposed fusion rule LRT-BER at the FC requires more power consumption due to the information decoding, which is not required for MRC, EGC, two-stage CV and LRT-CS.

## 6. Numerical Results

In this section, numerical results of the proposed LRT-BER for BPSK, DPSK and 2FSK modulations under the Rayleigh, Ricean and Nakagami-m fading envelope are given according to the performance evaluation in Section 5. Furthermore, the performance comparison with the existing MRC, EGC and LRT-CS rules is carried out under the N-P criterion. For the case of identical local detection performance indices, we first give the detection performance of the proposed fusion rule by using the closed-form system detection performance (Equations (29) and (30)). Then, the system detection performance of the proposed fusion rule is compared to the existing fusion rules.

### 6.1. Performance Evaluation of the Proposed LRT-BER Fusion Rule

**Figure 3:**
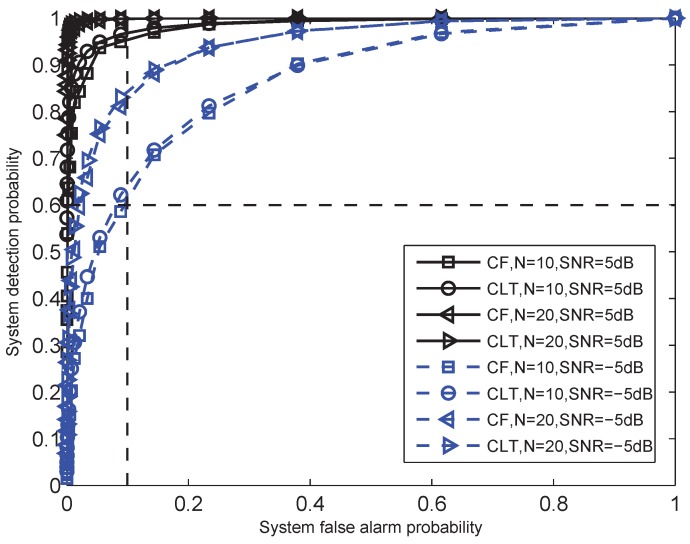
ROC curves for the likelihood ratio test (LRT)-bit error (BER) fusion rule based on central limit theorem (CLT) and closed-form (CF) performance evaluation with N = 10 and N = 20. Average received SNRs are assumed to be SNR = −5 dB and SNR = 5 dB. Each local detection index is assumed to be equal to each other ($P_{fa}$ = 0.1, $P_{d}$ = 0.6). BPSK modulation is used, and the Rayleigh fading envelope is assumed.

Figure 3 shows the receiver operating characteristic (ROC) curves of the proposed LRT-BER fusion rule in Equation (16). The false alarm probabilities and the detection probabilities are assumed to be identical for all sensors. In this experiment, we use $P_{fa}=0.1$ and $P_{d}=0.6$. We consider two different SNRs of the received signals, ${\bar{\gamma }}_{b}=-5$ dB and ${\bar{\gamma }}_{b}=5$ dB. The number of sensors is N=10 and N=20, respectively. BPSK modulation is used, and the Rayleigh fading envelope is assumed.

In Figure 3, both the closed-form performance evaluation in Equations (29) and (30) and the CLT approximation performance evaluation in Equations (31) and (32) are included. The label “CF” denotes the closed-form performance evaluation, and the label “CLT” denotes the CLT approximation performance evaluation. We can see that as the system false alarm probability increases, the system detection probability increases. Note that there is a gap between the CLT approximation and the closed-form performance evaluation under the same condition. This is because the CLT approximation performance evaluation is based on the assumption with a large number of sensors. However, for the setup of Figure 3, there is only 10 or 20 sensors. It can be observed that when the number of sensors increases, such a gap decreases. Furthermore, the gap under the SNR $\gamma_{b}=-5$ dB is smaller than that under the SNR ${\bar{\gamma }}_{b}=5$ dB. Besides, the detection performance of a single sensor system has been marked in the intersect of the two lines $P_{d}=0.6$ and $P_{fa}=0.1$. The fusion system based on LRT-BER fusion rule can significantly enhance the system detection performance under the case of received SNR ${\bar{\gamma }}_{b}=5$ dB. However, for ${\bar{\gamma }}_{b}=-5$ dB and N=10, the fusion system only slightly outperforms a single sensor system. This is because the degradation due to the communication channel leads to larger transmission error between sensors and the FC.

**Figure 4:**
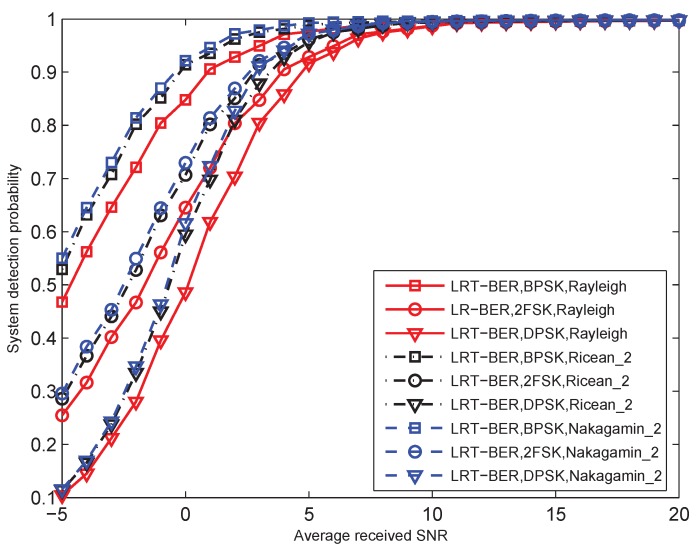
Detection probability of the LRT-BER fusion rule as a function of different received SNRs for BPSK, 2FSK and DPSK modulations with N = 20. Rayleigh fading, Ricean fading with factor K = 2 and Nakagami-m fading with m = 2 are considered. $P_{fa}=0.1$, $P_{d}=0.6$, and $P_{\mathrm{FA}}=0.01$.

**Figure 5:**
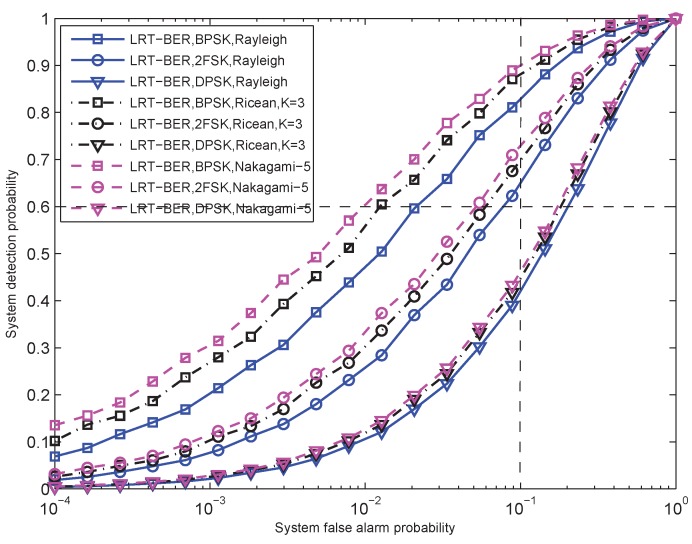
ROC curves for the LRT-BER fusion rule based on CF closed-form performance evaluation with N = 20. The average received SNR is ${\bar{\gamma }}_{b}=-5$ dB. Each local detection index is assumed to be equal to each other ($P_{fa}$ = 0.1, $P_{d}$ = 0.6). The system alarm probability is $P_{\mathrm{FA}}$ = 0.01.

To examine the relationship between the detection performance of the proposed LRT-BER Equation (16) and the physical layer specifications (modulation, reception mode and the channel characteristics), several experiments are given. Figure 4 gives the system detection probability as a function of SNR. The system false alarm probability is $P_{\mathrm{FA}}=0.01$. The number of sensors is N=20. The closed-form performance evaluation is used. In terms of the statistic characteristics of the flat and slow fading channel, Rayleigh fading, Ricean fading with factor K=2 and Nakagami-*m* fading with m = 2 are considered. Figure 5 describes the ROC curves. The false alarm probability is $P_{\mathrm{FA}}=0.01$, and the SNR is set to ${\bar{\gamma }}_{b}=-5$ dB. Other parameters are the same as they are in Figure 4.

From these two figures, it can be observed that the LRT-BER fusion rule with coherent BPSK modulation performs better than that with the other modulations for the identical statistic characteristics of the channel. This is because the BER of the BPSK modulation is smaller than that of 2FSK and DPSK modulations, which can be derived from Equations (7), (9) and (10). For identical modulation, the LRT-BER fusion rule with the Nakagami-2 assumption outperforms that with the Ricean-2 and Rayleigh assumption. The fusion rule with Rayleigh fading assumption exhibits the worst detection performance. This behavior can be explained easily. The Rayleigh fading assumption is the worst case, since the line of sight component of the received signal is not included. In Figure 5, we also give the detection performance of a single sensor system, which is marked as the intersection of the dashed lines. We can find that the detection performance of the proposed fusion rule with BPSK and 2FSK can be enhanced by fusing. However, when the DPSK modulation is employed, the fusion system does not provide enhanced performance. This is caused by the large transmission error of DPSK modulation, which is also derived from Equations (7), (9) and (10). From the above results, we can conclude that the non-ideal transmission channel can deteriorate the system detection performance and even exhibits worse performance than a single sensor.

### 6.2. Performance Comparison with the Other Fusion Rules

**Figure 6:**
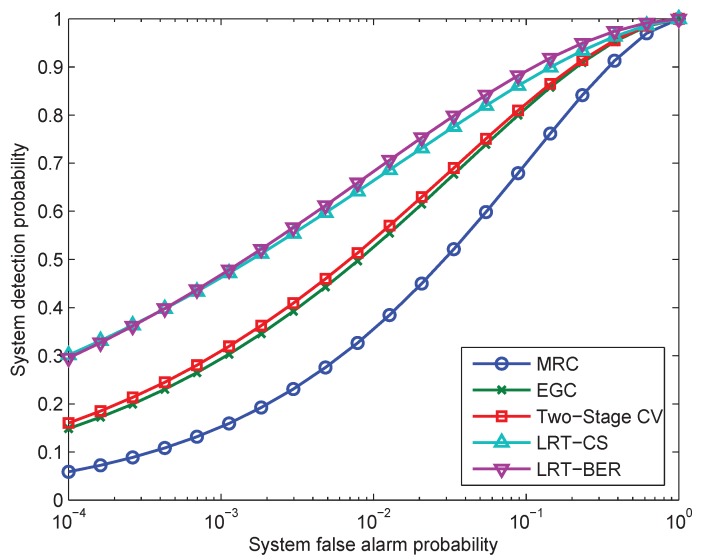
Comparison of ROC curves for LRT-BER, maximum ratio combiner (MRC), equal gain combiner (EGC) and LRT-channel statistics (CS) with six sensors, ${\bar{\gamma }}_{b}=5$ dB, $P_{fa}=0.1$, $P_{d}=0.6$. BPSK modulation and Rayleigh fading assumption are considered.

In this experiment, the detection performance of MRC, EGC, two-stage CV and LRT-CS is compared to the proposed LRT-BER fusion rule. Figure 6 gives the ROC curves of these fusion rules. The number of sensors is N=6. We assume that the local detection performance indices are equal to each other ($P_{fa}=0.1$ and $P_{d}=0.6$). The received SNR is set to ${\bar{\gamma }}_{b}=5$ dB, BPSK modulation and Rayleigh fading are considered. Since there is no closed-form performance evaluation for MRC and LRT-CS fusion rules proposed in [20], here the CLT approximation performance evaluation is employed. It can be observed that LRT-BER and LRT-CS exhibit a small deviation and perform better than MRC, EGC and two-stage CV. The performance of the MRC is worse than that of the other fusion rules.

**Figure 7:**
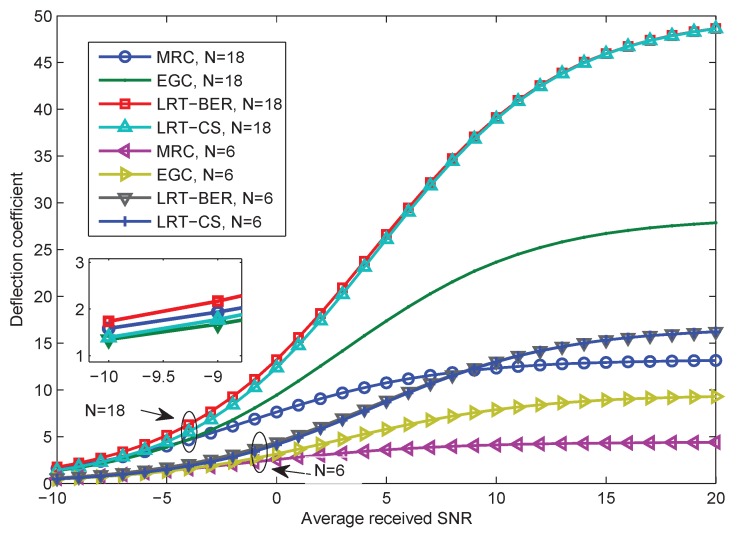
Deflection coefficient of MRC, EGC, LRT-CS, two-stage CV and LRT-BER statistics with six and eighteen sensors, respectively, $P_{fa}=0.1$, $P_{d}=0.6$. BPSK modulation and the Rayleigh fading assumption are considered.

In addition to the CLT approximation, the deflection coefficient in Equation (43) for the mentioned fusion rules is given in Figure 7. A zoom-in display of the deflection coefficient under the low SNR scenario is also included. The local detection performance indices are assumed to be identical for all sensors. In this experiment, we use $P_{fa}=0.1$ and $P_{d}=0.6$. BPSK modulation is employed. The number of sensors is N=6 and N=18, respectively. Figure 7 shows that the EGC method that requires the minimum amount of information performs better than the MRC under a wide range of received SNR. The only exception is that MRC slightly outperforms EGC under very low SNR (e.g., SNR <−5 dB). Furthermore, LRT-CS and the proposed LRT-BER present a small deviation. This is consistent with the results in Figure 6.

**Figure 8:**
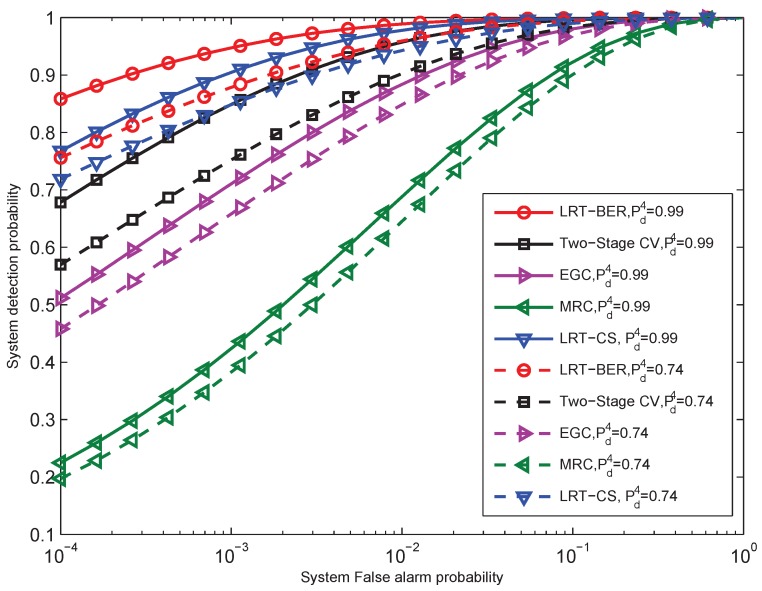
ROC curves of LRT-BER, MRC, EGC and LRT-CS statistics with different local detection probabilities and different received SNRs by using the CLT approximation. The local false alarm probability is $P_{fa}=0.1$. BPSK modulation and the Rayleigh fading assumption are considered.

In practice, sensors have different local detection performance. Furthermore, the received SNRs are not identical. Thus, we examine the detection performance of MRC, EGC, two-stage CV, LRT-CS, as well as the proposed LRT-BER fusion rule under the case of different local detection performance and different SNRs. Figure 8 gives the ROC curves for N=10 sensors by using the CLT approximation performance evaluation. The solid lines are the detection performance of the mentioned fusion rules with the setting of local detection probability $P_{d}=\left[0.43,0.75,0.68,0.74,0.75,0.32,0.75,0.68,0.70,0.70\right]$. In order to illustrate the system performance improvement caused by one sensor, the case with the fourth sensor local detection probability $P_{d}\left(4\right)=0.99$ (the local detection probability of Sensor 4 changes from 0.74 to 0.99) is also included in Figure 8. The dashed lines give the results with such case. The average SNRs are ${\bar{\gamma }}_{b}=\left[5.6,8.0,6.0,8.5,6.8,10.0,5.0,5.0,5.1,5.1\right]$ dB. The local false alarm probabilities are assumed to be equal to each other, which are set to $P_{\mathrm{fa}}=0.1$. BPSK is employed, and Rayleigh fading is assumed.

From the figure, we can see that when the local detection probabilities, as well as the received SNRs are not equal to each other, the proposed LRT-BER fusion rule outperforms significantly the other fusion rules. The detection performance of LRT-CS is better than MRC, EGC and two-stage CV. Furthermore, the MRC fusion rule presents the worst detection performance. Further, when one of the sensors improves the detection probability from $P_{d}\left(4\right)=0.74$ to $P_{d}\left(4\right)=0.99$, the improvement of the system detection probability $P_{\text{D}}$ (the difference between the solid line and the dashed line in Figure 8) due to the increasing of local detection probability of one sensor is larger than that of LRT-CS. Thus, we can concluded that the proposed fusion rule LRT-BER is more robust than the LRT-CS.

## 7. Conclusions

The problem of decision fusion for distributed detection under the non-ideal communication channel in WSNs is investigated in this paper. As the BER is able to characterize all uncertainties of the received data at the FC, an LRT-based fusion rule with non-ideal channel using BER is proposed. When the local detection performance indices of sensors are equal to each other, the closed-form system detection performance is derived. When the local detection performance indices of sensors are not identical, the CLT approximation and the deflection coefficient are used to describe the detection performance. Numerical results are presented to demonstrate the performance of the LRT-BER fusion rule. Furthermore, the performance of the proposed LRT-BER is compared to that of MRC, EGC, two-stage CV and LRT-CS. The proposed LRT-BER fusion rule provides the most robust detection performance compared to the mentioned fusion rules. However, the local detection performance indices and the received SNR are required for the proposed LRT-BER. The fusion rule under unknown local detection performance indices and SNR will be studied in our future work.
